# Iatrogenic Cushing Syndrome Secondary to Ritonavir-Epidural Triamcinolone Interaction: An Illustrative Case and Review

**DOI:** 10.1155/2014/849432

**Published:** 2014-05-07

**Authors:** Sapna Sadarangani, Melody L. Berg, William Mauck, Stacey Rizza

**Affiliations:** ^1^Division of Infectious Diseases, Mayo Clinic, 200 First Street SW, Rochester, MN 55905, USA; ^2^Ambulatory Care Pharmacy Services, Indiana University Health LifeCare Clinic, Indianapolis, IN 46202, USA; ^3^Division of Pain Medicine, Mayo Clinic, 200 First Street SW, Rochester, MN 55905, USA

## Abstract

HIV positive patients on ritonavir-containing antiretroviral therapy (ART) can develop iatrogenic Cushing syndrome (IACS) and adrenal insufficiency as a result of drug-drug interactions with inhaled or intranasal glucocorticoid therapy. Reports related to epidural triamcinolone injections are relatively uncommon but increasingly reported. We describe a 48-year-old woman with immunologically and virologically well-controlled HIV on ritonavir-based ART, who developed headache, dizziness, and candida and herpes simplex virus (HSV) ulcerative esophagitis 7 days after receiving an epidural triamcinolone injection for cervical radicular pain. Iatrogenic Cushing syndrome and relative adrenal insufficiency were suspected and proven. The patient's ART was changed to a non-HIV protease inhibitor- (PI-) containing program, her symptoms improved, and she did not require hydrocortisone replacement. In this paper, we review the literature on IACS and relative secondary adrenal insufficiency from epidural triamcinolone injections in HIV patients on ritonavir-containing ART regimens. A high index of clinical suspicion is needed for diagnosis. Prevention of drug-drug interactions by taking a thorough medication history for patients on ritonavir-containing ART regimens before prescribing any form of corticosteroid is crucial and effective and sustained interdisciplinary communication in the care of such patients.

## 1. Case

A 48-year-old HIV positive Caucasian woman, immunologically and virologically well controlled on a ritonavir-boosted protease inhibitor- (PI-) based antiretroviral therapy (ART) regimen, developed iatrogenic Cushing syndrome (IACS) and relative secondary adrenal insufficiency (SAI) following an epidural triamcinolone injection for cervical radicular pain.

The patient was diagnosed with HIV in 2008, which was acquired after a sexual assault. Her HIV viral load was 6400 copies/mL at time of diagnosis and CD4 T-cell count was 1125/*μ*L (31%) with a nadir CD4 T-cell count of 524/*μ*L in September 2010. Pretreatment HIV genotyping showed wildtype virus.

Screening for coinfections and sexually transmitted infections, including hepatitis C and syphilis, was negative. The HSV serum IgG was positive.

The patient had a nine-year history of chronic pain involving her right neck, suboccipital head, shoulder, low back, and lateral hip that had predated her HIV diagnosis. She had been followed by a pain medicine clinic and had received epidural and trochanteric bursa injections periodically for cervical radiculitis and trochanteric bursitis. She also had a history of tobacco use (30 pack years), remote history of illicit drug abuse with last use of IV cocaine in 1983. She had used alcohol heavily in the past but not within the last 10 years. Her psychiatric history included that of anxiety and depression/dysthymic disorder for which she was on low dose fluoxetine. Surgical history only included hysterectomy.

The patient had been followed clinically in the HIV clinic and had been in good health with CD4 T-cell count above 500. She was then randomized to be enrolled in the treatment arm of the (START) study—Strategic Timing of Antiretroviral Treatment. After enrollment, she was initiated on ART consisting of tenofovir-emtricitabine 300 mg/200 mg tablet daily, atazanavir 300 mg daily, and ritonavir 100 mg daily in June 2012. CD4 T-cell count was 596 and viral load was 39,500 copies/mL prior to therapy. She tolerated the antiretroviral regimen well; however, she developed a diffuse rash involving her trunk, arms, and thighs ten days after starting the ART program. She was evaluated by dermatology and her physicians in the HIV clinic, and the rash was not felt to be drug related. The rash eventually disappeared with no specific therapy and her HIV regimen was continued unchanged with no return of the rash.

The patient had been receiving cervical epidural triamcinolone injections for a year without incident prior to initiating ritonavir. For the six months prior to initiating ritonavir, the patient received two cervical epidural steroid injections and two trochanteric bursa injections with total dose of 60 mg triamcinolone and 6 mg dexamethasone. The last dose was 4 weeks prior to ritonavir initiation. Ten weeks after initiating ritonavir-based ART, the patient received a 40 mg triamcinolone epidural injection at C7-T1 and a 20 mg triamcinolone injection into the right trochanteric bursa to address her chronic neck and leg pain.

Seven days after the injection, she developed symptoms of headache, change in taste, and dizziness. She was advised to closely observe her symptoms and report any changes. Nine days later, she developed significant esophageal reflux symptoms despite having no prior history of gastroesophageal reflux disease. She was seen at her primary care internal medicine clinic and advised symptomatic therapy with an over-the-counter antacid.

Due to continued worsening of her symptoms, specifically worse epigastric pain, nausea, and esophageal reflux symptoms, the patient presented to the emergency department (ED) 5 days after developing esophageal reflux symptoms. Diagnostic workup included basic labs: white cell count of 12.2 × 10^9^/L with a normal differential (absolute lymphocyte count of 2150), hemoglobin 13.5 g/dL, platelets 263 × 10^9^/L, sodium 136 mmol/L, potassium 3.8 mmol/L, random glucose of 92 mg/dL, AST 22 U/L, bilirubin 2.6 (direct of 0.3) mg/dL, Cr 0.7 mg/dL, and lipase 30 U/L. An ultrasound of her hepatobiliary tree was normal. She was discharged from the ED with famotidine and aluminum/magnesium hydroxide suspension and advised to follow up with her primary care provider the following day.

At the follow-up visit at the primary care internal medicine clinic, an esophagogastroduodenoscopy (EGD) was arranged which revealed significant findings of scattered irregular ulcers 5–10 mm in size, from distal to mid esophagus which were biopsied. The proximal esophagus had scattered diffuse white exudates consistent with candida esophagitis. Ulceration covered 75% of the circumference of the gastroesophageal junction (5-6 mm). The biopsy findings from ulcerated mid and distal esophagus showed hyperplastic squamous esophageal mucosa and gastroesophageal junction-type mucosa with focal active chronic inflammation. The immunohistochemical staining of the mucosal tissue was positive for herpes simplex virus and negative for cytomegalovirus.

Four weeks following the epidural steroid injection and one week following the onset of esophageal reflux symptoms, the patient complained of “facial swelling” and insomnia. She had also gained 5 lbs and noted mood changes in the form of increased anxiety.

She was started on oral omeprazole and fluconazole therapy for 14 days for esophagitis. Due to ongoing esophageal symptoms, when HSV immunostain from EGD returned positive, a course of oral valacyclovir 1000 mg three times daily for 2 weeks was initiated. The patient's CD4 T-cell count at that time was 745 (25%) and serum HIV RNA viral load was detected but not quantifiable.

The patient's constellation of symptoms and normal CD4 T-cell count raised suspicion for IACS from interaction between triamcinolone injection and oral ritonavir therapy which would account for her relative immunodeficiency state despite an adequate CD4 T-cell number. A random morning serum cortisol was 3 mcg/dL (range 7–25 mcg/dL), and for an early morning specimen this signifies a lower serum cortisol value than physiologically expected.  Table 1 shows her ACTH (adrenocorticotropic hormone) stimulation test, also known as cosyntropin stimulation test and other relevant labs regarding her adrenal axis [1]. Her urine synthetic glucocorticoid screen detected significant triamcinolone 0.61 mcg/dL 4 weeks after her injection.

The patient began to complain of orthostatic dizziness particularly in the mornings; her orthostatic blood pressure and pulse rate (albeit in afternoon) were normal. She remained euglycemic throughout this time. Follow-up ACTH stimulation test showed an intact response.

Following completion of valacyclovir and fluconazole treatment courses, the patient's esophageal symptoms improved by 80% but did not completely resolve. A decision was made to increase the omeprazole dose and due to continued risk of medication interactions, the antiretroviral therapy program was changed to a non-PI based regimen, tenofovir-emtricitabine, and raltegravir.

Synthetic glucocorticoid screen did not show detectable triamcinolone levels, and a follow-up 24-hour urine cortisol was measured as 12 mcg/24-hour period 3 weeks following change in the ART program.

The score on the Drug Interaction Probability Scale assessing possible interaction between triamcinolone and ritonavir was 8, translating to a probable interaction [2]. No points were given for rechallenge of the drug (triamcinolone), and the dose also was not increased in this case as this was a one-time occurrence.

The patient's clinical course slowly improved and returned to baseline approximately 3 months after the triamcinolone injection, without requiring hydrocortisone replacement. She was advised not to have any further epidural triamcinolone injections without proper approval by her HIV specialist or endocrinologist.

## 2. Review of Other Cases

Our patient had significant morbidity as a result of HIV protease inhibitor and glucocorticoid interactions. We have reviewed the literature and report all available published experience of the interaction between ritonavir and triamcinolone resulting in IACS and secondary adrenal insufficiency.

Ritonavir, a potent inhibitor of the cytochrome P450 (CYP) 3 A4 isoenzyme, is used to increase therapeutic levels of other PIs (e.g., lopinavir, atazanavir, darunavir), thereby allowing for lower or less frequent dosing of the active PI. However, this portends numerous drug interactions with medications from various other classes including HMG Co-A reductase inhibitors, phosphodiesterase inhibitors, antiarrhythmics, and corticosteroids [3].

There have been numerous case reports on interaction of inhaled or intranasal fluticasone with ritonavir resulting in IACS and secondary adrenal insufficiency. There have been fewer but increasing number of recent case reports on the interaction between triamcinolone given as an epidural injection and ritonavir.

Foisy et al. published a review of 25 cases (15 adult and 10 pediatric) of adrenal suppression and Cushing's syndrome as result of an interaction between ritonavir and inhaled fluticasone [4]. The mean inhaled fluticasone dose was 992 mcg/day (range 500–2000 mcg/day) in adult patients and 455 mcg/day (range 200–1000 mcg/day) in pediatric patients. The majority of cases occurred with high doses of fluticasone and therefore the authors recommended great caution in giving inhaled fluticasone higher than 400 mcg/day in children and higher than 1000 mcg/day in adults on ritonavir based antiretroviral based programs.

There are fewer case reports on IACS and secondary adrenal insufficiency in context of triamcinolone and ritonavir.  Table 2 summarizes these cases [5–15]. Most patients presented with both IACS and SAI. More severe manifestations occurred in some patients including hyperglycemic hyperosmolar state [7], as well as avascular necrosis of the hip [8]. One patient had a rather delayed diagnosis with the fat distribution changes thought of as due to lipodystrophy [11]. The lowest dose of triamcinolone administered was a one-time 40 mg injection, and the highest was cumulative dose of 240 mg of triamcinolone given as 3 injections of 80 mg dose each. Seven of these cases needed hydrocortisone replacement. Four of these cases had received 80 mg or higher dose of triamcinolone, and the other three had received a single time 40 mg triamcinolone dose.

### 2.1. Clinical Presentation

The clinical manifestations of IACS as a result of ritonavir use vary greatly. The symptoms may be as subtle as change in weight distribution/weight gain that may be mistaken for lipodystrophy and the diagnosis may therefore be delayed potentially for months [11]. The more severe presentations reported include hypertension, glucose intolerance to the point of hyperglycemic hyperosmotic state, metabolic syndrome, and avascular necrosis resulting in significant morbidity [5–15]. Other severe presentations reported include steroid induced myopathy and herpes zoster reactivation due to secondary immune deficiency [14]. Our patient had a dramatic change in weight distribution and a relative immune deficiency state from prolonged systemic steroid exposure resulting in severe esophagitis (with candida and HSV esophagitis). Ramanathan et al. also described a patient who presented with symptoms of similar severity, although our patient had more severe esophagitis [6].

The presentation of relative secondary adrenal insufficiency may also be variable, ranging from fatigue to overt dizziness and hypotension. A high index of suspicion should be used for any of these symptoms in a patient on ritonavir and receiving any source of corticosteroids and taking a thorough drug history is crucial.

### 2.2. Diagnosis

When there is a clinical suspicion of HIV PI and glucocorticoid interactions, a patient's adrenal axis needs to be assessed in a systematic manner. An early morning random cortisol level should be obtained as well as an ACTH level. An ACTH stimulation test can be used to confirm adrenal axis suppression from exogenous steroids. A synthetic glucocorticoid screen is also beneficial in demonstrating the source of exogenous excess steroids. These may need to be repeated at more than one time point depending on patient's clinical course, especially if changes to the antiretroviral therapy regimen are made and/or symptoms are not improving with time as expected.

### 2.3. Management

Most patients in the literature had spontaneous recovery of symptoms and recovery of adrenal axis in a matter of months, ranging from 2 to 8 months depending on severity of the initial presentation and dose of triamcinolone used once the HIV PI or the glucocorticoid is stopped. Some patients required hydrocortisone replacement during the recovery phase. The need for hydrocortisone replacement appears to depend on the degree of suppression of the hypothalamus-pituitary-adrenal (HPA) axis (based on cosyntropin stimulation test) and is temporary until the HPA axis recovers. Ramanathan et al. showed the elimination half-life of triamcinolone acetonide was prolonged as much as 170-fold when coadministered with ritonavir [6]. Recommended steroid replacement is at a low physiological dose of 10–20 mg a day of hydrocortisone.

The first step, however, is to remove the source of exogenous corticosteroids and/or the ritonavir causing the medication interaction.

When possible, replacing the ritonavir-boosted PI regimen with another antiretroviral agent with no CYP3A4 inhibition such as a non-nucleoside transcriptase inhibitor, chemokine receptor 5 (CCR5 receptor) antagonist, or an integrase inhibitor (specifically raltegravir or dolutegravir), should be considered, as we did with our patient. However, choices may be limited in highly ART treatment experienced patients. In addition, it is not yet known whether cobicistat, currently used to boost elvitegravir concentrations via CYP3A inhibition mechanism, may also have a similar interaction and therefore would not be a good substitution for ritonavir-based regimen with respect to this particular drug-drug interaction.

Enteral or parenteral exogenous corticosteroids may be prescribed for various pain conditions across every subspecialty. The management of acute or chronic pain requires patient education as well as coordination of care with those prescribing ritonavir and those who may be offering exogenous corticosteroids. The literature describing the specific ritonavir and triamcinolone interaction is limited and not widely appreciated, but not unique to other corticosteroids. Alternative pain management options should be explored when a patient is on a boosted PI regimen.

## 3. Background/Discussion of Mechanism

Cytochrome P450 is a major pathway for drug metabolism via the liver. It is largely responsible for transferring lipid-soluble agents, through a process of oxidation, hydrolysis, or reduction, to water-soluble agents that are easier for the body to eliminate. Ritonavir is known to be a potent inhibitor of a particular isoenzyme of this metabolism pathway, CYP3A4 [16]. CYP3A4 is involved in the metabolism of over half of all commonly used medications [17]. In fact, it is because of its effect of inhibition of CYP3A4 that ritonavir continues to be included as an essential component of many antiretroviral regimens. All of the PIs used for treatment of HIV undergo metabolism, to some degree, by CYP3A4 and, therefore, use of these medications with ritonavir results in increased concentrations and prolonged half-life of the active PIs. This allows for reduced pill burden or decreased dosing frequency which may help improve adherence [18].

Although drug interactions with ritonavir and PIs are beneficial, the potent inhibition of CYP3A4 by ritonavir can have detrimental effects on other medications, particularly if the interaction potential goes unrecognized. In the case of our patient, an unrecognized interaction between ritonavir and a corticosteroid, triamcinolone, resulted in adrenal suppression. Glucocorticoids, including triamcinolone, undergo metabolism via CYP3A4 [19].

Therefore, use of ritonavir with glucocorticoids can result in increased exposure to the glucocorticoid and increased potential for side effects including HPA axis suppression, osteoporosis, and osteonecrosis.

Glucocorticoids differ with regards to potency and elimination half-life [19–25]. As mentioned previously, all undergo some degree of metabolism via the CYP3A4 enzyme. Hydrocortisone, methylprednisolone, and triamcinolone are the three corticosteroids used most commonly for injection. Triamcinolone and methylprednisolone have lower potency than hydrocortisone and longer half-life. All three agents have similar half-lives. The impact of these parameters on pharmacodynamics effect of these corticosteroids in the body following epidural injection is not clearly established. Systemic absorption of intra-articular injections has been shown to be dependent on the corticosteroid used, type of preparation, dose, number of injections given, and location of injection [26]. Triamcinolone has been shown to be absorbed into soft tissues from intra-articular and periarticular injections for 2-3 weeks [27]. There have been reported cases of Cushing syndrome in patients following triamcinolone injections using doses of 40 mg triamcinolone for pain, in the absence of other drugs that may cause drug-drug interactions thereby exponentially increasing systemic steroid levels [28].

## 4. Conclusion

The above case illustrates the importance of being cognizant of drug-drug interactions for HIV patients who are on ritonavir-containing regimens. This is particularly important in situations when the medication interactions may not have been well described or medications are administered in nontraditional methods, such as via inhalation or neuraxial injections.

In this particular case, the corticosteroid was documented through a separate surgical electronic record compared to other prescribed office based medications including this patient's ART. Also, at that time, this drug-drug interaction between triamcinolone and ritonavir was not as well described and did not appear in drug-interaction databases. Taking a thorough medical and medication (including over-the-counter medications) at each visit with HIV patients is imperative, as well as being vigilant in instances involving chronic pain conditions treated with injectable steroids for potential drug-drug interactions. This drug-drug interaction will not necessarily be readily recognized by the proceduralist administering injectable corticosteroids thus necessitating good communication between providers.

A similar concern for glucocorticoid-ART interaction is also present with cobicistat containing ART regimens although there is more evidence in the literature for ritonavir containing ART regimens.

Ongoing education of pharmacists, providers involved in care of these patients with emphasis on counselling and interdisciplinary communication between various HIV caregivers, and other specialists as well as patients themselves is important in prevention of such drug-drug interactions and morbidity. A high index of clinical suspicion is needed.

Furthermore, use of a universal medication ordering system would help identify such drug interactions, even when the medications were ordered in completely different departments, and prevent these potential complications.

If IACS and secondary relative adrenal insufficiency are suspected, as in this illustrative case, prompt assessment of steroid axis is key, as well as consideration of changing ART regimen, when possible, and a multidisciplinary knowledgeable approach to chronic pain for patients on boosted-PI regimens.

## Figures and Tables

**Table 1:** Assessment of the patient's adrenal axis at various time points following triamcinolone injection.

	Four weeks and 2 days after injection	Five weeks and 3 days after injection	Nine weeks and 2 days after injection	Fourteen weeks after injection and about 3 weeks after change in ART regimen
Cortisol 0 min (mcg/dL)	—	3.4	4.3	—
Cortisol 15 min following administration of cosyntropin) (mcg/dL)	—	8.0	—	—
Cortisol 30 min following administration of cosyntropin (mcg/dL)	—	13	21	—
Cortisol 45 min following administration of cosyntropin (mcg/dL)	—	14	—	—
Cortisol 60 min following administration of cosyntropin (mcg/dL)	—	—	26	—
Urine synthetic glucocorticoid screen	Triamcinolone 0.61 mcg/dL (range <0.10)	—	Negative	—
24 h urine cortisol	—	—	—	12 mcg/24 hours (range 3.5–45)

The criterion for expected serum cortisol on the standard high dose ACTH stimulation test is a minimum value 18 to 20 mcg/dL before or after ACTH injection [1].

**Table 2:** Summary of other cases.

Reference/author journal	Case	HAART regimen	Injection (TCA)	Clinical presentation	Hydrocortisone replacement	Time to recovery
Yombi et al. Clin Rheumatol 2008 [5]	54 yo woman	3TC/DDI/lopinavir-ritonavir	40 mg (knee)	IACS Hypertension SAI	20 mg daily	8 months
	56 yo man	D4T/AZT/indinavir-ritonavir	40 mg (cervical)	IACS SAI	10 mg daily	4 months
	49 yo woman	3TC/DDI/lopinavir-ritonavir	40 mg (shoulder)	IACS SAI	None	5 months

Ramanathan et al. [6] CID 2008	35 yo man	Tenofovir-emtricitabine/lopinavir-ritonavir	60 mg and then 80 mg (L spine)	IACS Hypertension Esophageal reflux	—	4 months

Danaher et al. [7] Orthopedics 2009	44 yo man	Ritonavir based regimen	80 mg (hip)	HHS-ICU admission IACS SAI	Unknown	Unknown

Dort et al. [8] AIDS Research and Therapy 2009	41 yo man	Tenofovir-emtricitabine, atazanavir-ritonavir	80 mg twice (epidural)	IACS Hypertension AVN hip (at 11 months)	None	6 months
	42 yo woman	Tenofovir-emtricitabine, atazanavir-ritonavir	40 mg (shoulder)	IACS	30 mg daily (short)	2 months

Levine et al. [9] J Am Acad Dermatol 2011	41 yo woman	Lamivudine, tenofovir, atazanavir-ritonavir	60 mg IM (topical steroid unresponsive nonspecific dermatitis)	IACS SAI	None	6 months

Albert et al. [10] Am J Med Sciences 2012	58 yo woman	Tenofovir-emtricitabine, fosamprenavir-ritonavir	Epidural dose is not mentioned	IACS SAI	None	2 months

Grierson and Harrast [11] Am Acad PMR 2012	47 yo woman	Tenofovir-emtricitabine, atazanavir-ritonavir	80 mg 3 occasions (epidural L spine)	“Lipodystrophy” DM Metabolic syndrome IACS SAI	20 mg daily (extended taper)	“Several months”

Fessler et al. [12] Pain Physician 2012	42 yo man	Tenofovir-emtricitabine, atazanavir-ritonavir	80 mg (lumbar epidural)	Hypertension Acne (on back)	None	3 months
	47 yo woman	Abacavir, lamivudine, darunavir-ritonavir → changed to abacavir, lamivudine, unboosted fosamprenavir upon dx	80 mg 2 occasions (lumbar epidural) Also used inhaled fluticasone/salmeterol inhaler for asthma for 5 days	Weight gain Emotional lability IACS Hypertension Oral candidiasis	None	10 weeks (improved)

Maviki et al. [13] Skeletal Radiology 2013	39 yo woman	Tenofovir-emtricitabine, darunavir-ritonavir	40 mg 2 occasions (right L5 nerve root)	IACS Oral candidiasis SAI	Hydrocortisone “maintenance”	8 months
	47 yo man	Tenofovir-emtricitabine, lopinavir-ritonavir	80 mg (subacromial, subdeltoid bursa)	IACS Hyperglycemia Weight gain SAI	Hydrocortisone “maintenance”	6 months

Schwarze-Zander et al. [14] Infection 2013	35 yo woman	Tenofovir-emtricitabine, saquinavir-ritonavir → changed to tenofovir-emtricitabine with raltegravir	6 times 20 mg weekly (L5-S1 periradicular)	IACS Hypokalemia SAI Steroid-induced myopathy Acute herpes zoster (4 week later)	Hydrocortisone 15 mg/daily-tapered at 8 months Comment: also needed potassium replacement for hypokalemia	8 months

Hall et al. [15] Int J STD AIDS 2013	53 yo woman	Darunavir-ritonavir Raltegravir	40 mg (left shoulder)	Hyperglycemia (worse than usual for her controlled DM) Hypertension (worse than prior) IACS Anxiety	None Comment: needed initiation of insulin for DM previously controlled by metformin	3 months

Sadarangani et al.	48 yo woman	Tenofovir-emtricitabine, atazanavir-ritonavir → changed to tenofovir-emtricitabine with raltegravir	40 mg triamcinolone epidural injection and 20 mg triamcinolone injection into right trochanteric bursa	Severe esophagitis (erosive, as well as candida and HSV esophagitis) IACS Relative SAI Mood changes-anxiety Weight gain	None Comment: needed course of fluconazole and valacyclovir as well as extended course therapy with proton pump inhibitor for esophagitis	3-4 months

HHS: hyperglycemic hyperosmolar state,

3TC: lamivudine,

DDI: didanosine,

D4T: stavudine,

AZT: zidovudine, and

AVN: avascular necrosis.
